# Evaluation of Antibody Response and Adverse Effects following Heterologous COVID-19 Vaccine Booster with mRNA Vaccine among Healthcare Workers in Indonesia

**DOI:** 10.3390/vaccines11071160

**Published:** 2023-06-26

**Authors:** Gatot Soegiarto, Bagus Aulia Mahdi, Laksmi Wulandari, Karin Dhia Fahmita, Satrio Tri Hadmoko, Hendra Ikhwan Gautama, Dewi Prasetyaningtyas, Muhammad Edwin Prasetyo, Pujo Prawiro Negoro, Nur Arafah, Dewajani Purnomosari, Damayanti Tinduh, Dominicus Husada, Ari Baskoro, Deasy Fetarayani, Wita Kartika Nurani, Delvac Oceandy

**Affiliations:** 1 Division of Allergy and Clinical Immunology, Department of Internal Medicine, Faculty of Medicine, Universitas Airlangga—Dr. Soetomo General Academic Hospital, Surabaya 60132, Indonesia; ari.baskoro@fk.unair.ac.id (A.B.); deasy-f@fk.unair.ac.id (D.F.); 2 Postgraduate School, Master Program on Immunology, Universitas Airlangga, Surabaya 60132, Indonesia; 3 Department of Internal Medicine, Faculty of Medicine, Universitas Airlangga—Dr. Soetomo General Academic Hospital, Surabaya 60132, Indonesia; bagus.aulia.mahdi-2018@fk.unair.ac.id (B.A.M.); karin.dhia.fahmita-2017@fk.unair.ac.id (K.D.F.); satrio.tri.hadmoko-2017@fk.unair.ac.id (S.T.H.); hen.ikhwan.gautama-2017@fk.unair.ac.id (H.I.G.); dewi.prasetyaningtyas-2019@fk.unair.ac.id (D.P.); mohammad.edwin.prasetyo-2018@fk.unair.ac.id (M.E.P.); pujo.prawiro.negoro-2019@fk.unair.ac.id (P.P.N.); nur.arafah-2018@fk.unair.ac.id (N.A.); wita.kartika@fk.unair.ac.id (W.K.N.); 4 Department of Pulmonology and Respiratory Medicine, Faculty of Medicine, Universitas Airlangga—Dr. Soetomo General Academic Hospital, Surabaya 60132, Indonesia; laksmi.wulandari@fk.unair.ac.id; 5 Department of Histology and Cell Biology, Faculty of Medicine, Public Health and Nursing, Universitas Gadjah Mada, Yogyakarta 55281, Indonesia; d.purnomosari@ugm.ac.id; 6 Department of Physical Medicine and Rehabilitation, Faculty of Medicine, Universitas Airlangga—Dr. Soetomo General Academic Hospital, Surabaya 60132, Indonesia; damayanti.tinduh@fk.unair.ac.id; 7 Department of Child Health, Faculty of Medicine, Universitas Airlangga—Dr. Soetomo General Academic Hospital, Surabaya 60132, Indonesia; dominicus.husada@fk.unair.ac.id; 8 Division of Cardiovascular Sciences, Faculty of Biology Medicine and Health, Manchester Academic Health Science Centre, University of Manchester, Manchester M13 9PT, UK; 9 Department of Biomedical Science, Faculty of Medicine, Universitas Airlangga, Surabaya 60132, Indonesia

**Keywords:** COVID-19, vaccine, booster, comorbidity, antibody response, healthcare worker, adverse event, mRNA vaccine, inactivated viral vaccine

## Abstract

*Background*: The administration of the third (or booster) dose of COVID-19 vaccine is important in maintaining protection against SARS-CoV-2 infection or the severity of the disease. In Indonesia, health care workers (HCWs) are among the first to receive a booster dose of the COVID-19 vaccine. In this study, we evaluated the antibody response and adverse events following heterologous booster vaccine using mRNA-1273 among HCWs that were fully vaccinated with inactivated viral vaccine as the priming doses. *Methods*: 75 HCWs at Dr. Soetomo General Hospital in Surabaya, Indonesia, participated in this study. The level of antibody against the SARS-CoV-2 receptor binding domain was analyzed at 1, 3, and 5 months following the second priming dose and at 1, 3, and 5 months after the booster dose. *Results*: We found a significantly higher level of antibody response in subjects receiving a booster dose of the mRNA-1273 vaccine compared to those receiving an inactivated viral vaccine as a booster. Interestingly, participants with hypertension and a history of diabetes mellitus showed a lower antibody response following the booster dose. There was a higher frequency of adverse events following injection with the mRNA-1273 vaccine compared to the inactivated viral vaccine, although the overall adverse events were considered minor. *Conclusions*: A heterologous booster dose using mRNA vaccine resulted in a high antibody response; however, participants with hypertension and diabetes mellitus displayed a lower antibody response.

## 1. Introduction

The national vaccination program for Coronavirus Disease 2019 (COVID-19) in Indonesia started on 13 January 2021 with health care workers (HCWs) being one of the first groups of people receiving the vaccine. The success of the vaccination program among HCWs is crucial to protecting them from the risk of contracting COVID-19 from their work. Recent reports have shown a considerable high acceptance of the COVID-19 vaccine in people from low- and middle-income countries (LMIC) [1]. However, other studies have demonstrated a variability among HCWs in Asia and Africa in terms of willingness to take the COVID-19 vaccine [2,3,4]. Our own observation of HCWs in two major hospitals in East Java, Indonesia, indicated a high uptake of COVID-19 vaccination, i.e., more than 80% have received two doses of vaccine within the first 3 months after vaccine roll-out [5].

The inactivated viral vaccine was the main type of vaccine used by the Indonesian authorities at the beginning of vaccination. One of the inactivated SARS-CoV-2 vaccines, CoronaVac, which was used in the national vaccination program, showed a very good level of protection against severe COVID-19, hospitalization, and mortality [6,7]. However, recent published data have demonstrated a waning of antibody levels and protection following COVID-19 vaccination over time [8,9,10,11]. Observations on people who were vaccinated with the mRNA vaccine have suggested a decrease in serum antibody levels by 38% in each following month [9]. This might lead to an increase in the incidence of COVID-19 breakthrough infections, as reported elsewhere [11]. Despite a number of observations on mRNA vaccines, there were fewer reports regarding waning immunity and the reduction of antibody levels in people vaccinated with inactivated viral vaccines.

The importance of the COVID-19 vaccine booster is evident. It has been demonstrated that a third (booster) dose enhanced both humoral and cellular immunity regardless of the type of the priming doses and the type of the booster dose itself [12]. Moreover, the side effects of booster doses seem acceptable, as shown by the relatively non-severe adverse effects in people receiving booster vaccines, as reported in a previous publication [12].

We have evaluated the serum antibody levels against SARS-CoV-2 among HCWs in East Java, Indonesia, following vaccination using inactivated virus. We observed a significant increase in the serum antibody level [13]. However, we discovered that participants with hypertension displayed lower serum antibody levels compared to those with normal blood pressure [13]. 

In this present study we performed a follow up analysis of the same cohort of HCWs as described above, who received a booster dose of SARS-CoV-2 vaccine at 5 months following the priming dose. Most of the participants received the mRNA-1273 vaccine (Moderna) as a booster dose following recommendations from the Indonesian Health Authority, while a few of them opted to receive a booster dose using an inactivated viral vaccine (CoronaVac). Here we assessed the antibody response, the adverse effects of the vaccine, and the association of the antibody response with hypertension following the third (booster) dose of vaccines in our cohort of HCWs.

## 2. Materials and Methods

### 2.1. Study Participants

This is a follow-up observation on our previously reported study [13]. We recruited non-pregnant health care workers (HCWs) at Dr. Soetomo General Hospital in Surabaya, Indonesia. Apart from chronic underlying conditions such as diabetes, hypertension, and allergic diseases, the participants did not have any other diseases at the beginning of the study. All of the participants were tested for the presence of antibodies against SARS-CoV-2 before the start of the study. Participants with a detectable level of serum IgG against SARS-CoV-2 RBD before the first dose of vaccination and those who contracted COVID-19 (confirmed by PCR test) during the course of the study were excluded from the study since any infection with SARS-CoV-2 virus before or during the study might affect the serum antibody level against SARS-CoV-2 virus and hence confound the data. From 101 individuals who were originally involved in the study, 8 had positive serum IgG against SARS-CoV-2 before the priming dose of vaccination, 18 contracted SARS-CoV-2 infection during the course of the study, and 2 dropped out due to unwillingness to undergo follow-up examination. Thus, 75 participants fulfilled the criteria and were included in the analysis. 

### 2.2. COVID-19 Vaccination

All participants received two doses of the inactivated SARS-CoV-2 vaccine (CoronaVac) as the priming doses. An analysis of serum samples was conducted at 1, 3, and 5 months after the second dose of vaccination. All participants were offered a booster dose with either the mRNA-1273 vaccine (Moderna) or the inactivated viral vaccine (CoronaVac) at 5–6 months following the second dose. Serum samples were again analyzed at 1, 3, and 5 months after the booster dose.

### 2.3. Serology Assay

We used a commercially available kit (Elecsys Anti-SARS-CoV-2 S, Roche Diagnostics, Mannheim, Germany) to examine the level of IgG against the SARS-CoV-2 receptor-binding domain (RBD) in the serum samples. We followed the protocol as recommended by the manufacturer.

### 2.4. Demographic and Adverse Events Data Collection

At the beginning of the study, we interviewed participants regarding demographic data and the presence or history of comorbidities (i.e., diabetes mellitus, cardiovascular disease, and allergic disease). Blood pressure was also measured at the beginning of the study and during follow-up visits. During the follow-up visits, participants were also asked about the presence of vaccine adverse reactions.

### 2.5. Statistical Analysis

The serum IgG level is presented as geometric mean titres and 95% confidence intervals (CI). To analyze the difference in IgG level between booster vaccination with mRNA vs. inactivated viral vaccine, we used a non-parametric multiple comparisons (Mann–Whitney U) test. The same test was also used to analyze the effects of comorbidities on the antibody response. A *p* value less than 0.05 was considered statistically significant. We used GraphPad Prism ver. 9 (GraphPad Software, LLC, Boston, MA, USA) to analyze the data. To control for the possible confounding effects of each comorbidity, a multivariate logistic regression analysis was performed with inclusion of histories of hypertension, diabetes mellitus, and cardiovascular diseases.

## 3. Results

### 3.1. Study Participants

This is a follow-up observation on our previously reported study [13]. However, in the present analysis, we only included HCWs who have never been infected with SARS-CoV-2 and have an undetectable level of serum IgG against the SARS-CoV-2 receptor binding domain (RBD) before the first dose of vaccination. Participants who contracted COVID-19 (confirmed by PCR test) during the course of the study and those who had baseline IgG levels against SARS-CoV-2 RBD were excluded from the study. A total of 75 HCWs who fulfilled these criteria were included in the analysis. The mean age of participants was 50.95 years old, and 60% of them were male. Hypertension was detected in 29.3% of participants. Some of the participants have a history of diabetes mellitus (21.3%), cardiovascular diseases (14.7%), and allergic diseases (42.7%) (Table 1).

### 3.2. Serum IgG Level against SARS-CoV-2 Receptor Binding Domain (RBD) 

**Figure 1:**
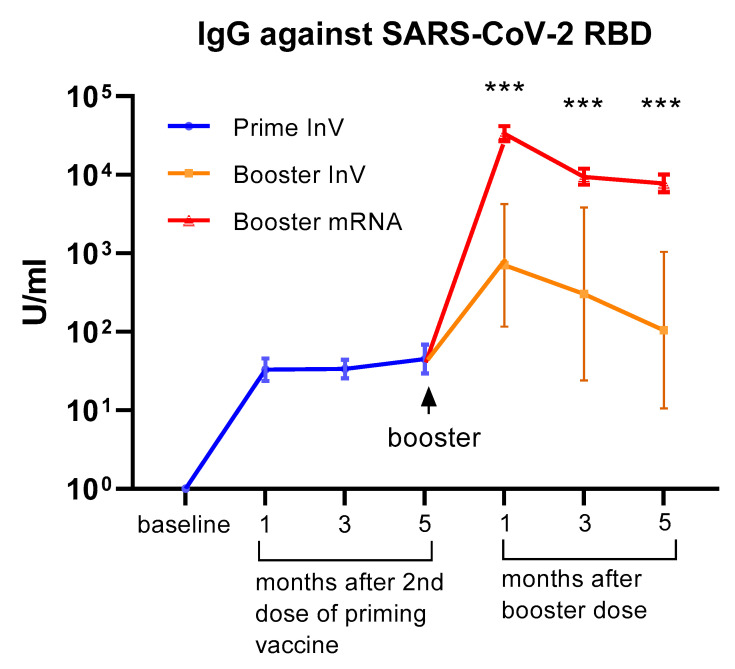
Serum antibody levels (IgG) against the SARS-CoV-2 receptor binding domain (RBD) at 1–5 months following priming (1st and 2nd doses) vaccination and at 1–5 months after the booster dose of COVID-19 vaccination. All of the participants (*n* = 75) received an inactivated viral vaccine for the priming dose. A total of 69 participants received the mRNA-1273 vaccine as a booster, whereas 6 participants received an inactivated viral vaccine as a booster. The data are presented as GMT and 95% CI. *** *p* < 0.001, multiple non-parametric test (Mann–Whitney U test), InV = inactivated viral vaccine.

All of the participants received inactivated viral vaccine (CoronaVac) as the priming doses (first and second doses). Of the 75 HCWs in our cohort, 69 of them received the mRNA-1273 vaccine (Moderna) as a booster (third dose), whereas 6 subjects opted to have an inactivated viral vaccine (CoronaVac) for the booster dose. We analyzed serum IgG levels against the RBD domain at 1, 3, and 5 months following the 2nd dose of priming vaccine and at 1, 3, and 5 months after receiving the booster dose. As shown in Figure 1, we observed a marked increase in serum IgG levels following priming doses. Interestingly, the level of serum IgG did not significantly decrease up to 5 months after the priming doses. A marked increase in antibody levels was observed in all participants following the booster dose. The level of serum IgG against SARS-CoV-2 seemed higher in participants receiving the mRNA-1273 vaccine compared to those who had an inactivated viral vaccine as a booster (Figure 1). Although the difference reached statistical significance, it needs to be interpreted cautiously since the number of subjects receiving boosters with inactivated vaccines was very low.

### 3.3. Antibody Response in Participants with Comorbidities

**Figure 2:**
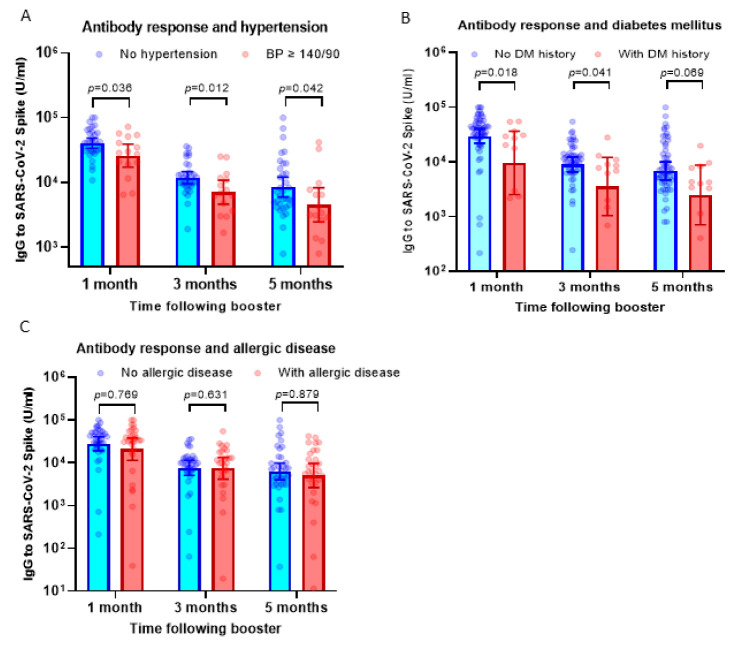
Antibody response following booster dose in participants with comorbidities. (**A**) Serum IgG levels against SARS-CoV-2 RBD in participants with hypertension (blood pressure/BP ≥ 140/90) compared to subjects with normal blood pressure. On average, the IgG levels of participants with hypertension were 30–43% lower compared to subjects without hypertension. (**B**) IgG levels in participants with a history of diabetes mellitus. On average, the IgG levels of participants with diabetes mellitus were 40–60% lower compared to subjects without hypertension. (**C**) IgG levels in participants with a history of allergic diseases. The serum IgG levels were comparable between subjects with and without allergic diseases. Multiple non-parametric tests (the Mann–Whitney U test) were used to compare the differences.

Our previous observation has suggested that subjects with hypertension display a lower antibody response against the priming doses of inactivated viral vaccine [13]. To understand if hypertension affected the response to the booster dose using the mRNA-1273 vaccine, we compared serum IgG levels between participants with hypertension (BP ≥ 140/90) and those without hypertension. Interestingly, we found a consistent finding that participants with high BP showed a lower antibody response following booster doses using the mRNA-1273 vaccine (Figure 2A).

The antibody response in participants with histories of diabetes mellitus (DM) and allergic diseases was also analyzed. Similar to participants with hypertension, we observed significantly lower antibody levels in subjects with a history of diabetes mellitus at 1–5 months following booster vaccination (Figure 2B). However, in contrast with the finding above, participants with allergic diseases displayed comparable levels of serum IgG following boosters compared to those without allergic diseases (Figure 2C). 

To analyze the possible confounding effect between these three comorbidities, we conducted a multivariate linear regression analysis. Data presented in Table 2, Table 3 and Table 4 suggested that hypertension showed the strongest association with serum IgG level post-booster vaccination and remained significantly associated with antibody response, in particular at 1 month and 3 months after booster vaccination (Table 2 and Table 3). History of diabetes mellitus showed a trend of significance at 1 month post-booster vaccination, whereas there was no significant association between history of allergic disease and serum IgG level. Overall, our finding showed that among co-morbidities, hypertension significantly influences the antibody response following a booster dose of the mRNA vaccine.

### 3.4. Adverse Reactions following Vaccination

**Figure 3:**
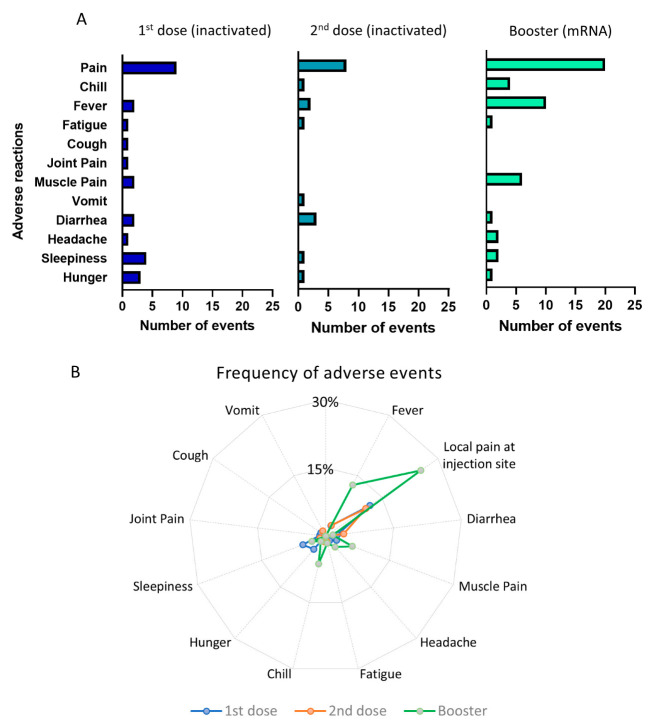
Adverse events following priming and booster doses of COVID-19 vaccination. (**A**) Number of adverse events following the 1st and 2nd doses of inactivated viral vaccine and booster doses of mRNA vaccine. (**B**) Radial graph showing the frequency of adverse events comparing the 1st, 2nd, and booster doses of vaccination.

The pattern of the adverse effects is depicted in Figure 3A,B. In total, there were 26 events of adverse reactions reported after first dose of priming vaccination, 18 reactions following the second dose of priming vaccine, and 48 reactions after the booster dose of vaccination. It is clear that booster injections using the mRNA-1273 vaccine triggered more frequent adverse effects than the priming doses using inactivated viral vaccine. The most frequent adverse effect following a booster dose was pain at the injection site, which was reported by 20 participants (25.4%), followed by fever (10 participants, 12.7%) and muscle pain (6 participants, 6.3%). The adverse effects of the first and second priming doses using inactivated viral vaccine were less frequent. For example, only 10–12% reported local pain following injection, whereas other side effects occurred in less than 5% of the participants. However, despite the higher frequency of adverse effects following booster vaccination, the majority of them were non-severe and transient. None of the participants required hospital treatment due to the side effects of the vaccine.

## 4. Discussion

The main finding of this study is that a heterologous booster dose of the mRNA-1273 vaccine induces a strong antibody response in individuals who have been vaccinated with an inactivated viral vaccine. This data is in line with previous reports and adds to the growing body of evidence showing the effectiveness of heterologous booster vaccination in enhancing antibody levels against SARS-CoV-2 [12,14,15,16]. Another important finding of our study is that participants with hypertension and a history of diabetes mellitus (DM) exhibited a lower antibody response following the booster dose compared to those with normal blood pressure or without DM history.

The effectiveness of mRNA vaccines as booster doses in enhancing antibody titers is evident. When compared to other platforms of COVID-19 vaccine, for example, adenovirus, recombinant protein, and inactivated viral vaccine, the mRNA vaccine showed superiority in terms of the level of antibody response [12]. Based on a study by Zhang et al., it was stated that the long-term antibody levels following vaccination with inactivated viral vaccine at 11–12 months post-vaccination are very low. Consequently, people who were vaccinated with an inactivated viral vaccine might still be susceptible to SARS-CoV-2 infection, although they have a lower severity of COVID-19 infection than those who were not vaccinated [17]. Our present data is in line with previous reports indicating a strong antibody response to the mRNA vaccine as a booster, regardless of the type of the priming vaccine. Importantly, we also found a significantly enhanced antibody response in participants who received inactivated viral vaccine as a booster dose, although it was at a significantly lower level than the response against mRNA booster vaccine. However, it is important to note that the number of subjects in this category was very small.

One novel finding of this study is that the antibody response against the booster dose is significantly lower in participants with hypertension (BP ≥ 140/90) and those with a history of DM compared to subjects without these comorbidities. Previous observations have indicated a reduction in antibody response following the COVID-19 vaccine in subjects with hypertension [13,18,19,20,21,22] and diabetes mellitus [21,22,23,24,25,26]. These phenomena were reported on subjects who received inactivated viral vaccines [13,18], mRNA vaccines [19,20,22,24] and adenoviral vaccine [25] as the priming doses. Our present data indicate that (i) subjects with hypertension and diabetes mellitus also showed reduced antibody responses following the third or booster dose, and (ii) the reduction of the response occurred following heterologous booster vaccination with mRNA vaccine. This further supports the idea that hypertension and diabetes mellitus may play a very important role in determining antibody responses against vaccination. Indeed, the association between hypertension, diabetes mellitus, and dysregulation of immune response has been widely documented previously [27,28], and our study has added to the line of evidence in terms of vaccine response. Further studies to delineate the precise mechanism are needed in order to better understand this phenomenon and find strategies to minimize the detrimental effect of high blood pressure in reducing vaccine response.

It is important to note that the median age of the participants was relatively high (57 years). Interestingly, the proportion of participants with hypertension in our cohort was lower compared to the prevalence of hypertension in the Indonesian population within the age group of 55–64 years old [29]. However, the prevalence of diabetes in our cohort seemed comparable to the prevalence in the general population at a similar age (19.6%) [29]. 

The other important finding of this study was that the antibody response to the booster dose declined over time. Our analysis suggested that the antibody titer at 5 months after the booster dose declined by more than 70% compared to the antibody titer at 1 month post-booster. Evidence showing the waning immunity following priming doses of COVID vaccine is accumulating [8,10], and this has become one of the main reasons for the importance of having a booster dose. Our data provide new evidence that the serum antibody level is also declining following a booster dose. We do not have evidence whether this decline will result in a reduction in protection since we did not assess the incidence of breakthrough infections in our cohort. The status of the cellular immunity, which is associated with protection against disease severity, also needs to be evaluated to understand if the protection following a booster dose is also reduced over time. However, it is important to note that recent observations suggested that people with a lower antibody response were more prone to breakthrough infections against the new Omicron variants [30].

In addition to antibody response, another important aspect that needs to be assessed is the effectiveness of controlling the incidence and severity of COVID-19. In our previous observation, we analyzed the incidence and severity of COVID-19 among a cohort of HCWs who received vaccination with an inactivated viral vaccine [5]. We assessed COVID-19 incidence and severity by comparing the period before and after the start of the national vaccination program. We found a significant reduction in COVID-19 hospitalizations in the period after vaccination compared to before vaccination. However, we still observed a higher incidence of infection in the fully vaccinated cohort at the time when a new variant circulated in the population [5]. The incidence of breakthrough SARS-CoV-2 infection due to a new variant (in the case of our cohort, the Delta variant) indicated that an appropriate strategy for booster vaccination is necessary to control COVID-19 incidence and severity in the future. Equally important, the use of multivalent COVID-19 vaccines should always be considered given the occurrence of multiple SARS-CoV-2 variants in Indonesia, as indicated by data in the GISAID database [31].

The mRNA vaccine has been widely associated with more side effects compared to other types of vaccines [12,32]. Our data concur with previous studies, in which we found more frequent adverse effects, both systemic and local, following vaccination using mRNA vaccine compared to the effects after vaccination with inactivated viral vaccine. However, it is important to note that most of the adverse effects in our cohort were temporary, and none of them required hospitalization. This indicates that the booster vaccination using mRNA vaccine is generally safe for people who have received inactivated viral vaccine before.

Our present study has several limitations. First, we only examined humoral responses by assessing serum IgG levels. As mentioned previously, cellular immunity is important in protecting against disease severity. Further studies are needed to understand whether cellular immunity is enhanced following a heterologous primary vaccine-booster combination of inactivated viral vaccine with mRNA vaccine and whether cellular immunity is waning over time following booster. Second, we only have a small number of participants in this study. It is important to note that a number of study subjects were excluded due to infection with the SARS-CoV-2 virus during the study period. We recruited infection-naïve subjects, and so some of the participants who had detectable antibodies at the beginning of the study were excluded. Another limitation was the possibility of asymptomatic SARS-CoV-2 infection among the participants following the priming doses, which might affect the level of serum IgG antibody. We did not perform an analysis of anti-nucleocapsid antibodies, which could detect asymptomatic infection, due to limited resources. However, we closely monitored for the presence of COVID-19 symptoms among all the participants and followed this up with a PCR test during the study, so the possibility of asymptomatic SAS-CoV-2 infection was probably minimal. The detection of anti-nucleocapsid antibody may also be useful in comparing antibody responses against inactivated virus vs. mRNA vaccines, since inactivated viral vaccines may contain N-proteins in addition to S-proteins, whereas mRNA vaccines only contain S-antigens. Studies to focus on this aspect are needed in the future.

It is clear that HCWs need to be protected against COVID-19 since they may have higher exposure to the virus due to possible contacts with patients. In our recent observation, we found a higher incidence of COVID-19 in medical staff (i.e., physicians and nurses) compared to non-medical hospital workers, such as administrative staff, during the first year of the COVID-19 pandemic in 2020 [5]. This is in line with other reports that showed a higher risk of healthcare workers contracting the SARS-CoV-2 infection [33,34,35,36]. This underlines the importance of giving healthcare workers appropriate protection, including protective clothing and booster doses of vaccines.

In summary, our findings show that heterologous booster doses using mRNA vaccine in a cohort of HCWs who received priming vaccine with inactivated virus induce a higher antibody response than homologous booster vaccine with inactivated SARS-CoV2. However, the level of serum IgG waned over time and participants with hypertension displayed a lower antibody response. Our data adds to the growing body of evidence showing the importance of the COVID-19 booster vaccination to improve protection against the disease. 

## Figures and Tables

**Table 1:** Characteristics of study participants.

	Participants Included in This Study (*n* = 75)
Sex	
Male	45 (60%)
Female	30 (40%)
Age at vaccination (y)	
Mean ± SD	50.95 ± 19.55
Median	57
Blood pressure	
Non-hypertension	53 (70.7%)
Hypertension (BP ≥ 140/90)	22 (29.3%)
History of diabetes mellitus	
No	59 (78.7%)
Yes	16 (21.3%)
History of cardiovascular diseases	
No	64 (85.3%)
Yes	11 (14.7%)
History of allergic diseases	
No	43 (57.3%)
Yes	32 (42.7%)

SD—standard deviation; BP—blood pressure.

**Table 2:** Multivariate regression analysis of serum IgG level at 1 month post-booster dose.

Variable	Regression Coefficient	*p* Value
**Hypertension**	**−0.235**	**0.05**
History off Diabetes Mellitus	−0.232	0.07
History of allergic disease	0.006	0.96

Variable with significant association is written in bold.

**Table 3:** Multivariate regression analysis of serum IgG level at 3 months post-booster dose.

Variable	Regression Coefficient	*p* Value
**Hypertension**	**−0.246**	**0.05**
History off Diabetes Mellitus	−0.185	0.16
History of allergic disease	0.114	0.38

Variable with significant association is written in bold.

**Table 4:** Multivariate regression analysis of serum IgG level at 5 months post-booster dose.

Variable	Regression Coefficient	*p* Value
Hypertension	−0.168	0.18
History off Diabetes Mellitus	−0.139	0.28
History of allergic disease	−0.060	0.64

## Data Availability

The datasets created and analyzed during this study are available from the corresponding authors upon reasonable request.
